# Enzyme characteristics of pathogen-specific trehalose-6-phosphate phosphatases

**DOI:** 10.1038/s41598-017-02220-2

**Published:** 2017-05-17

**Authors:** Megan Cross, Siji Rajan, Janine Chekaiban, Jake Saunders, Chloe Hamilton, Jeong-Sun Kim, Mark J. Coster, Robin B. Gasser, Andreas Hofmann

**Affiliations:** 1 Griffith Institute for Drug Discovery, Griffith University, Nathan, Queensland 4111 Australia; 2 Department of Chemistry, Chonnam National University, Gwangju, 61186 Republic of Korea; 3 Faculty of Veterinary and Agricultural Sciences, The University of Melbourne, Parkville, Victoria 3010 Australia; 4 Queensland Tropical Health Alliance, Smithfield, Queensland 4878 Australia

## Abstract

Owing to the key role of trehalose in pathogenic organisms, there has recently been growing interest in trehalose metabolism for therapeutic purposes. Trehalose-6-phosphate phosphatase (TPP) is a pivotal enzyme in the most prominent biosynthesis pathway (OtsAB). Here, we compare the enzyme characteristics of recombinant TPPs from five important nematode and bacterial pathogens, including three novel members of this protein family. Analysis of the kinetics of trehalose-6-phosphate hydrolysis reveals that all five enzymes display a burst-like kinetic behaviour which is characterised by a decrease of the enzymatic rate after the pre-steady state. The observed super-stoichiometric burst amplitudes can be explained by multiple global conformational changes in members of this enzyme family during substrate processing. In the search for specific TPP inhibitors, the trapping of the complex conformational transitions in TPPs during the catalytic cycle may present a worthwhile strategy to explore.

## Introduction

As opposed to organism-based drug screening approaches, where the entire pathogenic organism is probed in phenotypic or survival assays, protein-based strategies have the distinct advantage of providing insights into the molecular mechanisms of chemical effectors. Therefore, in order to develop novel therapeutic approaches, pathogen proteins identified as promising targets need to be appraised from molecular and structural viewpoints. The results from such an appraisal then provide a starting point for informed structure-based drug design.

An unprecedented wealth of genomic and transcriptomic sequence data are now publicly available for bacterial and parasitic pathogens. A promising avenue to identify novel drug targets thus involves a comparison of pathogen and host genomes, with the aim of finding genes in the pathogen that are distinct from those of the host (sometimes referred to as subtractive genomics). Ideally, such targets should be essential in the pathogen, i.e. are crucially important for its development and survival. Upon interference with or interruption of such targets, the viability and growth of a pathogen should be substantially impaired, thus ultimately leading to the clearance of the pathogen from the host. Additionally, an ideal target protein in a pathogen should not have an orthologue in the host, such that the possibilities of ‘cross-reactions’ of a specific chemotherapeutic with host proteins and pathways are reduced, helping to minimise side effects^1^.

The fact that trehalose is an essential oligosaccharide for many micro-organisms, but is neither required nor synthesised by mammalian cells, has previously attracted interest from researchers targeting the biosynthetic pathway of trehalose for chemotherapeutic intervention^2^. Trehalose (also known as mycose or tremalose) is a non-reducing disaccharide consisting of two glucose subunits with an α,α′-1,1′-glycosidic bond. This carbohydrate occurs in a wide range of species and is synthesised by bacteria, fungi, both lower and higher order plants and various invertebrates. Trehalose has arguably received most study in plants and fungi, where it has roles in development, abiotic stress tolerance, energy storage and the regulation of carbon metabolism^3^ and, thus, has implications for the global food supply. Studies of plants (reviewed in refs 4, 5) have demonstrated that modification of trehalose metabolism enables the engineering of plants with higher biomass content or increased abiotic stress tolerance.

Five different pathways of trehalose biosynthesis have been observed in prokaryotes, plants, fungi and non-vertebrate animals. Many eubacteria possess between two and four pathways, whereas invertebrates as well as fungi and plants only possess one pathway^6^. Of the five different pathways of trehalose synthesis, the only conserved pathway (OtsAB pathway) among plants, fungi and invertebrates, was first described for yeast^7^ and is regulated by the enzyme trehalose phosphate synthase (TPS), which catalyses the formation of trehalose-6-phosphate from UDP-glucose and glucose-6-phosphate. The phospho group is removed by trehalose-6-phosphate phosphatase (TPP) to yield trehalose^8, 9^. Knockdown of either the TPS genes (*tps-1*, *tps-2*) or the TPP gene (*gob-1*) in the free-living roundworm *Caenorhabditis elegans* showed that an accumulation of trehalose-6-phosphate, rather than the absence of trehalose, leads to a lethal phenotype^10^. Similarly, the blocking of otsB2 in *Mycobacterium tuberculosis* results in cell poisoning^11^. Notably, TPP is conserved in pathogenic species but absent from mammalian hosts; the enzyme thus fulfills all of the above criteria for a worthwhile drug target against nematodes and is validated in other species including mycobacteria.

Since drug discovery and development typically employ extensive studies of structure-activity relationships, an understanding of the mechanism of action of the target enzyme(s) is imperative. TPPs belong to the haloacid dehalogenase (HAD) family of phosphatases^12^. The HAD domain constitutes 20% of all human phosphatase domains and catalyses dephosphorylation of an extensive range of substrates. Structurally, this is achieved by highly conserved active site residues positioned within a Rossmann-like fold known as the core domain. In some family members, including TPPs, the core domain sequence contains an inserted cap domain, which may enclose the active site upon substrate binding^13^. Cap domains are linked to diversification within the family, and can be divided into three classes (C0, C1 and C2) based on structure and insertion position. In all HAD proteins, the core (and, where present, cap) domains are believed sufficient to achieve dephosphorylation and any additional domains are linked to functional diversity^14^. HAD phosphatases are magnesium-dependent and share a common mechanism that involves a nucleophilic attack by an aspartate, resulting in the formation of a phospho-aspartyl intermediate that is then hydrolysed by a water molecule in a second step, releasing phosphate and regenerating the catalytic nucleophile^13^. Based on a survey of mono-enzyme TPPs from a variety of pathogenic organisms, it has previously been suggested that these enzymes can be classified into three groups based on their structural topology (see also Supplementary Figure S2), thus distinguishing among enzymes from nematodes, mycobacteria, other eubacteria and archaea^15^.

In the present study, we compared the enzyme kinetics of three novel and two known mono-enzyme TPPs from important pathogenic organisms. From the nematode group, we investigated the TPPs of *Ancylostoma ceylanicum* (abbreviated as *Acey*), *Brugia malayi* (*Bmal*) and *Toxocara canis* (*Tcan*). *B. malayi* is the causative agent of lymphatic filariasis in humans while *A. ceylanicum* and *T. canis* are canid parasites that have recently become zoonotic. Human infections by both species are increasing^16–20^ and have severe consequences for hosts, particularly where worm migration into the subretinal space may cause blindness^21, 22^. For this reason, toxocariasis is prioritised by the Centers for Disease Control and Prevention for public health action^23^, with *A. ceylanicum* infection likely to follow as reports of the worm in humans continue to emerge^19^. From the bacterial groups, we selected the TPPs from *M. tuberculosis* (*Mtub*) (also called otsB2 or Rv3372), and from the bacterial group we selected the TPP from *Stenotrophomonas maltophilia* (*Smal*), an opportunistic pathogen associated with hospital-acquired infections^24, 25^. Our analysis shows that all enzymes possessed the expected phosphatase activity against trehalose-6-phosphate. We further identified a burst-like kinetic activity for all enzymes studied, which had varying but generally limited susceptibility to generic phosphatase inhibitors.

## Results and Discussion

### Expression and purification

For protein production, we adopted a practical and effective strategy based on synthetic genes utilising codon optimisation according to the expression organism. The synthetic genes encoding TPPs of *A. ceylanicum*, *B. malayi*, *T. canis*, *M. tuberculosis* and *S. maltophilia* were each ligated into an expression vector that contained an N-terminal hexa-His-tag and a tobacco etch virus (TEV) protease cleavage site. While recombinant *B. malayi* and *M. tuberculosis* TPPs had been expressed and purified previously^2, 26^, recombinant TPPs from *A. ceylanicum*, *T. canis* and *S. maltophilia* were produced here for the first time (Supplementary Figure S3).

The presence of successful folding and expected secondary structure in the novel TPP enzymes from *A. ceylanicum*, *T. canis* and *S. maltophilia* (as well as the known TPPs from *B. malayi* and *M. tuberculosis*) was verified by using far-ultraviolet circular dichroism spectroscopy (Supplementary Figure S4). The spectra indicated mixed α/β secondary structure elements, in agreement with the α/β nature of the Rossmann fold constituting the TPP core domain. In particular, the CD spectrum of the *Acey*-TPP construct confirms that the removal of three exons of the gene reported for the hypothetical protein Y032_0015g2804 (gb:EYC23728.1)^27^, as suggested earlier^15^, yields a folded gene product.

### Overall enzyme kinetics and steady state parameters

Previous studies highlighted that TPPs are highly specific enzymes and only process trehalose-6-phosphate as substrate with reasonable catalytic efficiency^10, 28–30^. Here, the wild-type enzymes *Acey*-TPP, *Bmal*-TPP, *Tcan*-TPP, *Mtub*-TPP and *Smal*-TPP were tested in a phosphatase kinetics assay using trehalose-6-phosphate as substrate. The amount of phosphate released was determined colorimetrically employing an endpoint assay based on phosphomolybdate/malachite green chemistry^31^.

**Figure 1:**
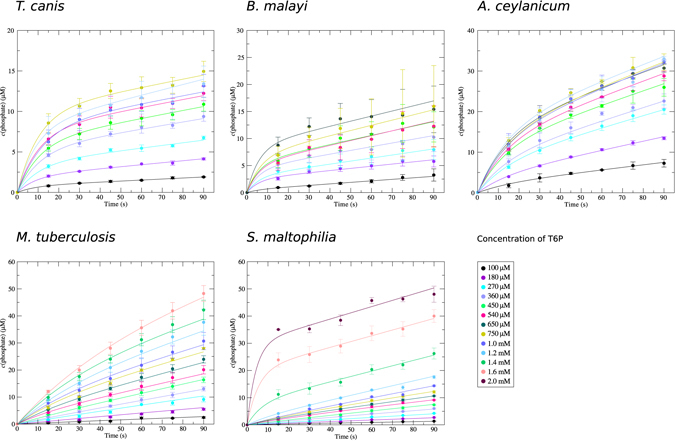
Enzyme kinetics. Time-dependent product formation catalysed by five TPPs from pathogenic organisms at varying initial substrate concentrations. Data points were acquired by quenching the reactions after the indicated incubation period. The data points represent the mean value of at least three independent experiments, and error bars indicate one standard deviation. The fits of individual datasets with the burst equation are shown as solid lines.

**Figure 2:**
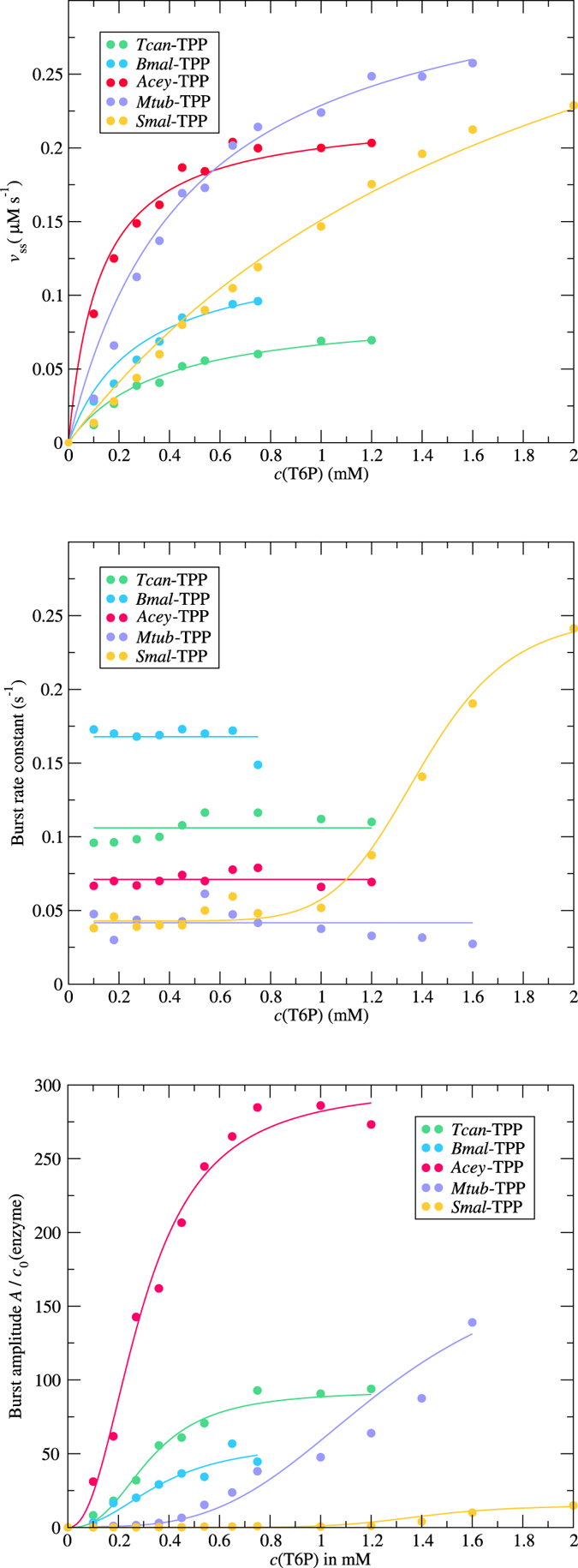
Analysis of steady state (**A**) and pre-steady state (**B**,**C**) kinetic parameters, obtained from fits of the burst equation to the raw data (Fig. 1). (**A**) Steady state kinetics. Data points show the steady-state velocity for different initial substrate concentrations. The solid lines represent fits of the Michaelis-Menten equation; for numerical results see Table 1. (**B**) The burst rate constants observed for varying initial substrate concentrations. For *Smal*-TPP, the data were fitted assuming a dose-response relationship (solid line); *EC*_*50*_: 1.39 mM, *width*: 7.8. (**C**) The burst stoichiometry, calculated as the quotient of burst amplitude and enzyme concentration, as observed for varying initial substrate concentrations. The solid lines represent fits assuming a dose-response relationship. The fit parameters were obtained as *EC*_*50*_ in mM: 0.325 (*Tcan*-TPP), 0.371 (*Bmal*-TPP), 0.297 (*Acey*-TPP), 1.25 (*Mtub*-TPP), 1.39 (*Smal*-TPP); *width*: 2.7 (*Tcan*-TPP), 2.3 (*Bmal*-TPP), 2.3 (*Acey*-TPP), 3.5 (*Mtub*-TPP), 7.8 (*Smal*-TPP). Note the agreement of the dose-response parameters of *Smal*-TPP with the correlation observed for the burst rate constant.

For all TPPs tested, the time-dependent plots of the amount of released phosphate showed bi-phasic progress with a clear upward curvature during the initial phase of the enzymatic reaction, indicating an initial high velocity of enzymatic turnover immediately after adding enzyme to substrate (Fig. 1). In the brief time preceding the steady state (pre-steady state), an enzyme binds and processes the first substrate molecules. If the release of the product is rate limiting, a burst phase is observed, during which the population of enzyme molecules becomes saturated with substrate^32^. At the end of the burst phase, the progress curve becomes linear (constant reaction rate) and the system enters the steady state phase. Accordingly, the raw data from the present study were fitted with a three-parameter burst equation, yielding the amplitude and rate constant for the burst phase, as well as the steady state rate. For an assessment of steady state kinetics, the rate was plotted against the substrate concentration to yield a Michaelis-Menten plot (Fig. 2A); the steady-state kinetics parameters are summarised in Table 1. A comparison of steady state kinetic parameters of *Bmal*-TPP^26^ and *Mtub*-TPP^33, 34^ reported previously and those determined in the present study shows general agreement. The individual TPP enzymes from different species are characterised by *K*_*M*_ values of between 0.1 and 2.0 mM and catalytic efficiencies of between 10^2^ and 10^4^ M^−1^ s^−1^. Compared with the limiting diffusion rate constant of approximately 10^9^ M^−1^ s^−1^, the catalytic efficiencies of these enzymes are low to moderate, owing to low turnover numbers (*k*_*cat*_).

**Table 1:** Enzyme parameters of steady state kinetics for different TPPs.

		*K*_*M*_ mM	*k*_*cat*_ s^−1^	*k*_*cat*_/*K*_*M*_ M^−1^ s^−1^	Temperature °C	Reference
Nematodes	*Acey*-TPP	0.12	4.7	38 × 10^3^	25	This study
	*Bmal*-TPP	0.28	0.9	3.3 × 10^3^	25	This study
		0.36	24	67 × 10^3^	25	26
	*Tcan*-TPP	0.35	0.9	2.6 × 10^3^	25	This study
	*Asuu*-TPP^a^	0.23	3.6	16 × 10^3^	25	60
Mycobacteria	*Mtub*-TPP [otsB2, Rv3372]	0.48	1.7	3.5 × 10^3^	25	This study
		0.6	n. d.		25	33
		0.48	2.8	5.8 × 10^3^	37	34
		0.50	10	20 × 10^3^	25	60
Bacteria	*Smal*-TPP	2.0	0.23	0.11 × 10^3^	25	This study
	*Sboy*-TPP^b^	0.69	16	23 × 10^3^	25	60
	*Styp*-TPP^c^	0.31	6.2	20 × 10^3^	25	60

The table summarises the numerical results of fitting the Michaelis-Menten equation to the steady state data shown in Fig. 2A and comparison with kinetics data from the literature.

n. d.: no data; ^a^ *Ascaris suum*; ^b^ *Shigella boydii*; ^c^ *Samonella typhimurium*.

### Pre-steady state parameters

Burst kinetics are most commonly observed in cases where there is an accumulation of enzyme-bound intermediates or products^35–42^. The observation of burst behaviour with TPP enzymes is thus in agreement with the assumed formation of a covalent phospho-aspartate intermediate in HAD phosphatases^12, 43, 44^.

The three-parameter burst equation allows the determination of two pre-steady state parameters, the burst amplitude and the rate constant for the burst phase. For the TPP enzymes tested in this study, the burst rate constant appeared to be invariant with respect to substrate concentration (Fig. 2B). The notable exception in the panel of tested enzymes was *Smal*-TPP, for which the burst rate constant increased with the concentration of substrate, and the correlation coincided with that observed for the substrate dependency of the burst amplitude.

**Figure 3:**
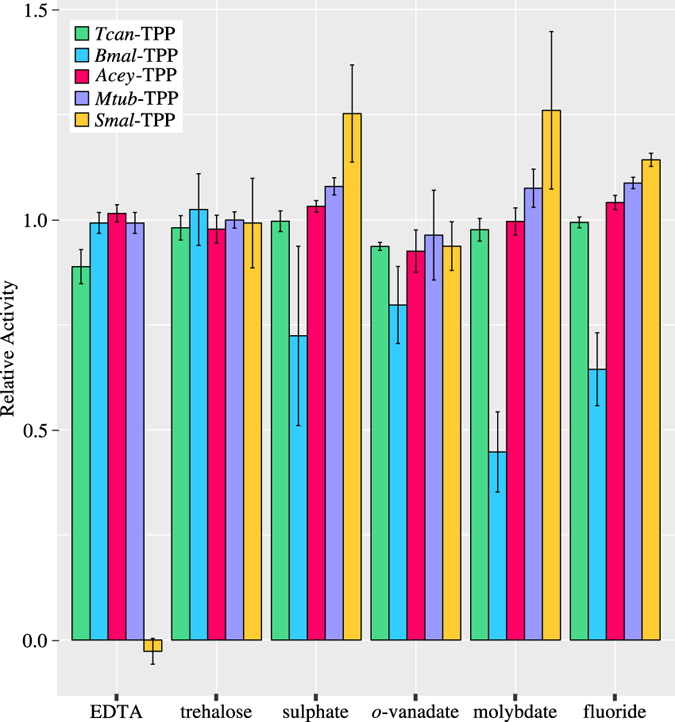
Inhibition of five TPPs from pathogenic organisms. The effects of the generic inhibitors EDTA, sulphate, *o*-vanadate, molybdate and fluoride, as well as the product from the enzymatic reaction, trehalose, were tested at 1 mM final concentration. Relative activity was calculated as the ratio of phosphate concentration determined in an endpoint assay of reactions in the presence and absence of inhibitor. The bar heights indicate the mean value of at least three independent experiments, and the error bars represent one standard deviation.

**Figure 4:**
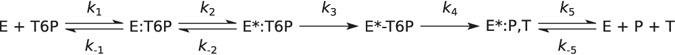
Kinetic scheme of the elementary steps in the TPP-catalysed hydrolysis of trehalose-6-phosphate. E – open enzyme conformation; E* – closed enzyme conformation; T6P – trehalose-6-phosphate; T – trehalose; P – inorganic phosphate.

The burst amplitude (Fig. 2C), normally expected to attain a maximum of one product molecule per catalytic site, reached values that were considerably greater than the enzyme concentration and was thus super-stoichiometric, indicating that the occurrence of the burst behaviour involved a process occurring over the period of several enzyme turnovers. Such behaviour leads to a reduction of the rate with time, as is the case for product inhibition or enzyme isomerisation, for example, rather than a ‘normal’ burst due to slow product release^32^. Enzymatic reactions of TPPs carried out in the presence of various concentrations of trehalose did not show any inhibitory effects (Fig. 3), thus ruling out product inhibition. Therefore, these findings suggest that the apparent burst observed in the enzyme kinetics of TPPs is due to a complex scheme of elementary reactions (Fig. 4) involving domain movements^30^.

In the first step, trehalose-6-phosphate binds to the open form of the enzyme, and it is assumed that the occurrence of productive interactions between enzyme and substrate cause the cap domain to rotate towards the core domain (step 2), thus forming a closed substrate-bound form. Nucleophilic attack by an active site aspartate side chain in the third step leads to a covalent intermediate that is further hydrolysed into the two product molecules (step 4). Finally, the enzyme is required to remove the cap from the core domain in a conformational change (step 5). It can reasonably be assumed that the steps involving domain movements (steps 2 and 5) proceed much slower than the chemistry steps (3 and 4). These multiple processes occur in a population of enzyme molecules in a non-synchronised fashion. Additionally, it is possible that individual enzyme molecules undergo a conformational change between open and closed states in the absence of substrate molecules. Such non-synchronised conformational changes will lead to a decrease in the overall rate of the enzymatic reaction of a population of molecules.

### Enzyme inhibition

Owing to the exquisite substrate specificity of trehalose-6-phosphate phosphatases, there are currently no known specific inhibitors of these enzymes. In previous studies, some selected generic phosphatase inhibitors have been tested against *Mtub*-TPP^29^ and *Bmal*-TPP^26, 28^, and transition state analogues have been used to trap the enzymatically competent conformation of TPP enzymes from *Thermoplasma acidophilum* ^45^ and *Candida albicans* ^30^. The only non-generic compounds reported to have moderate inhibitory effects on any TPP are the phosphoglycolipid antimycobacterials diumycin and flavomycin (moenomycin A) which were found in of *M. tuberculosis* and *M. smegmatis* ^2^.

TPPs from the present study were tested for inhibition utilising a panel of generic phosphatase inhibitors (see Fig. 3), including EDTA, sulphate, *o*-vanadate^46^, molybdate and fluoride^47^. Substantial inhibitory effects were only observed on *Smal*-TPP (with EDTA) and *Bmal*-TPP (with molybdate or fluoride). As a general observation, *Bmal*-TPP was the most and *Acey*-TPP the least susceptible of the enzymes tested in this study. Clearly, the absence of any notable effects of EDTA on most TPPs indicates that magnesium coordination of these enzymes is generally very strong, owing to the highly negatively charged coordination sphere provided by the multiple aspartate side chains. The abolished enzymatic activity of *Smal*-TPP in the presence of 1 mM EDTA is a notable exception.

In order to evaluate the possibility of product inhibition, the effects of added trehalose were also tested. None of the TPPs in this study showed any significant change in activity and the possibility of product inhibition can therefore be excluded.

## Conclusions

Based on the recent classification of mono-enzyme TPPs from bacterial and parasitic pathogens of animals, representative TPPs from each of the three groups were chosen here for the characterisation of enzyme kinetics. Whilst TPPs from *B. malayi* and *M. tuberculosis* have previously been investigated, this study is the first report of recombinant TPPs from *A. ceylanicum*, *T. canis* and *S. maltophilia*. The nematodes *T. canis* and *A. ceylanicum* are parasites of canids and felids but can also involve other animals and humans as paratenic hosts^48^. Importantly, *A. ceylanicum* is one of the commonest hookworms infecting humans^49^, and *T. canis* infection has been linked to allergic disorders in humans, such as urticaria, chronic pruritus and/or asthma^17^. The Gram-negative bacterium *S. maltophilia* is ubiquitously present in the environment and is naturally resistant to many broad-spectrum antibiotics^50^. In immuno-compromised patients, this bacterium can cause latent pulmonary disease of major clinical concern^51^, and has been associated with high morbidity and mortality^52^.

Together with other compatible solutes, including polyols, glucosylglycerol and certain amino acids and their derivatives, trehalose acts as an osmoprotectant and thus contributes to the protection of organisms against osmotic stress^53^. Interestingly, while most organisms utilise a spectrum of different osmolytes, *S. maltophilia* relies entirely on trehalose for osmoprotection^54^. In this context, it is interesting that *Smal*-TPP is the enzyme with the lowest catalytic efficiency in the panel of TPPs investigated here. The slow turnover number and comparatively large *K*_*M*_ value may be an adaptation to a physiological setting where constitutively large amounts of trehalose-6-phosphate are present. In mycobacteria, trehalose not only acts as an osmo- and thermoprotectant, but is also an important constituent of the unusual mycobacterial cell wall where it is present as lipo-conjugates in the form of cord factor (trehalose 6,6′-dimycolate) and sulfolipids (acylated trehalose-2′-sulphate derivatives), allowing functional rehydration of the membrane structure after desiccation^55^. Additionally, these cell wall metabolites have also been associated with tissue damage resulting from infection with pathogenic mycobacteria^56^. As a precursor of these cell wall components, trehalose is critical for mycobacterial growth^57^. In contrast, in nematodes, trehalose is the major sugar in the circulating haemolymph, and functions as an energy reserve, in addition to its thermo- and osmoprotective roles^58, 59^.

The prominence of the OtsAB pathway in pathogenic organisms and the absence of TPP from vertebrate hosts has not only drawn attention to these enzymes as potential drug targets, but also raises the hope that broad-spectrum chemotherapeutics could be developed. It is an imperative pre-requisite for the rational design of drugs against specific targets to understand their molecular and enzymatic mechanisms. In this study, we compared the enzymatic characteristics of TPPs from selected pathogens and provide a rigorous characterisation of their enzymatic behaviour. All members of the enzyme panel studied here displayed a burst-like kinetic behaviour. Due to the formation of a covalent intermediate with the substrate during turnover and a subsequent rate-limiting release of the enzyme-bound product, a stoichiometric burst behaviour would not be unexpected for TPPs. However, the observation of super-stoichiometric burst amplitudes leads to the conclusion that the burst appearance is a repercussion of multiple conformational changes at a global scale required by the enzyme for substrate capture, catalytic turnover and product release.

The fact that generic phosphatase inhibitors as well as close substrate analogues^60^ possess only limited inhibitory effects emphasises the need for further drug discovery studies and novel design approaches. The importance of conformational transitions in the TPP enzyme mechanism suggest that molecules trapping dysfunctional conformations of TPPs may be worthy of further explorations.

## Materials and Methods

### Protein expression and purification

Codon-optimised expression constructs of the TPP genes from *A. ceylanicum* (edited sequence of gb:EYC23728.1; see Supplementary Information S1), *B. malayi* (gb: XM_001893174.1; gene: *Bma*-*gob*-1), *T. canis* (gb:KHN76157.1), *M. tuberculosis* (gb:CP007299) and *S. maltophilia* (gb:CCH13862) were obtained from GenScript (USA), ligated into the vector p11 (obtained from The Biodesign Institute, Arizona State University, USA) via *Nde* I and *Bam* HI restriction sites, resulting in protein constructs with an N-terminal fusion peptide (MGSSH_6_SSGRENLYFQGH).

Expression and purification was performed according to the protocol published previously^15^. Purified protein samples (see Supplementary Figure S3) were dialysed against 100 mM NaCl, 1 mM MgCl_2_, 1 mM DTT and 20 mM TRIS (pH 8.0) and concentrated by ultrafiltration using an Amicon Ultra cartridge (Merck, Kilsyth, VIC, Australia) with 30 kDa cutoff. CD spectroscopy of purified proteins indicated the presence of the expected secondary structure elements (Supplementary Figure S4). The final purified non-tagged proteins were subjected to mass spectrometry to validate their identity by MS fingerprinting (see Supplementary Table S5).

### Enzyme kinetics

The substrate α,α′-trehalose-6-monophosphate was synthesised in-house as published previously^15^. Phosphatase activity of purified recombinant proteins was assessed after removal of the N-terminal fusion peptides. The purified recombinant proteins were tested at 0.25–2.5 μM in a buffer solution comprising of 20 mM TRIS (pH 7.5) and 100 mM NaCl, as well as varying concentrations of trehalose-6-phosphate (0.1–2 mM). Reactions were set up in a total volume of 350 μl in 96-well plates at room temperature, started by addition of 0.05–36 μM enzyme, and stopped after various time points (0.5–15 min) by addition of 100 μl of BIOMOL^®^ Green reagent (Enzo Life Sciences, New York, USA). Following incubation for 15 min, the absorbance at 620 nm was measured using a Biotek plate reader. All reactions were set up in triplicate in 96-well plates (Corning, Sigma Aldrich, NSW, Australia) and control experiments in the absence of enzyme were used to correct for background absorbance.

Absorbance data were converted to molar concentration of phosphate using a calibration function that was re-determined for every new batch of BIOMOL^®^ Green. All data fitting was performed with the software SDAR^61^. To model the burst behaviour, the raw kinetics data were fitted with the following equation

$$c({\rm{phosphate}})=A\cdot ({\rm{1}}-{{\rm{e}}}^{-{k}_{b}\cdot t})+{v}_{{\rm{steady}}{\rm{state}}}\cdot t,$$

where *A* is the burst amplitude, *k*_*b*_ the burst rate constant and *v*_steady state_ the steady state rate^32^.

### Enzyme inhibition

To test the inhibitory properties of compounds, end-point assays with fixed substrate concentration and reaction time were used. Enzyme activity in the absence of compounds was assessed in 50 μl reaction mixtures with 2.5 μM TPP protein in assay buffer (100 mM NaCl, 20 mM TRIS, pH 7.5). All compounds tested for potential inhibition were added at a final concentration of 1 mM and the mixtures were incubated for 5 minutes before reactions were initiated by the addition of trehalose-6-phosphate (200 μM final concentration). Reactions were allowed to proceed for 15 min before quenching with 100 μl of BIOMOL^®^ Green reagent. After an incubation period of 15 min for colour development, the absorbance at 620 nm was measured using a Biotek plate reader. All reactions were set up in triplicate in 96-well plates and control experiments in the absence of enzyme were used to correct for background absorbance.

## Electronic supplementary material

Supplementary Information
