# Supplementary material for: Enzyme characteristics of pathogen-specific trehalose-6-phosphate phosphatases
Source: Sci Rep. 2017 May 17;7:2015. doi: 10.1038/s41598-017-02220-2 (PMC5435700; doi:10.1038/s41598-017-02220-2)
Supplement: Supplementary file 1 — Supplementary Information [file 41598_2017_2220_MOESM1_ESM.pdf]

Enzyme characteristics of pathogen-specific trehalose-6-phosphate phosphatases

Megan Cross, Siji Rajan, Janine Chekaiban, Jake Saunders, Chloe Hamilton, Jeong-Sun Kim, Mark J Coster, Robin B Gasser & Andreas Hofmann

Supplementary Information

S1 Edited cDNA sequence of *Ancylostoma ceylanicum* trehalose-6-phosphate phosphatase

The full length sequence of this gene was obtained by aligning gb:EYC23728 and gb:GASF01016442[1].

```
atgtcatcgggaagcgaagaggctcagtgtgtcgaggccttcaagcgcatgttgtacacgatgcaaaatgtgcgtcgtac
gatcgtggaacgtattctaaacgagtgtgaagtggacgaagctgatgtagatgctctcgacaaagccttacaagagttga
cggactcgcgaacatctggagaaatgcgacacatctctacgccagcggccaacttccccatcaatatcagggacgaaatt
cgtggactacggaaagactgcgaatttctgcatcgtctgtcgaaggtcacatctaaatcaccaatgattgagaatgctct
ggatcagatccagttggatacgattttggctccgtatcatccagaaagtccgaagaaatttgaggaagagctgcaagacg
cggaacggtttcttatggatttcgttgattccgcatattcgggtgtaaagccgctgcttgtaacagactgggacggcacc
atgaaggactactgctcgcagtacgccacgaatttgcagccagtgtacagtgccgtcgttatgggtcgatttgctgagct
tttcacccgagctactgctgtgcttactgctggacctttgaggggtcctggcattttggatctgacagctctgccaataa
acggtccagttttgtttagcggttcctggggtcgtgaatggtggctgcgtggacgccgtgttgtacacgaggatggaatc
tccgaagagggttttgatgcaatcggtcgacttagtgatgagatgaccgatttgctcgaggacagcagcttcgcgcagtt
cgcgctcgtcggcagcggtgtgcagagaaaggtggatcgcctcactttgggtgtgcaaaccgtatttggtcatgtacctc
tcgaactggtcgtgagatacattgacgctatcaaggagagaattcatcgtgttgatccgaacaatgctaatctcgtcttg
gagaactcttcgcccttggagattgaggtctgtgcgcataattcaggagctgtatggaataaaggtgatggggtggcatc
attgattgaatctctacacgattcattgaaaaatggaaaagttttggtggctggtgacaccacaagtgatctgccgatgc
ttcaacatgcagtttccgagaatccagatggtgtcatggcactattcgttggtgctggcgaatcgttacgtgagtccgta
caatcgattgtcggcgatgaatccagagtctgttttgtgtcttgtccggatgtggtgcacgcggcttttgcccgtgtact
ggccgccaaagttgagttggactag
```

S2 Structure-based amino acid sequence alignment

Structure-based amino acid sequence alignment of trehalose-6-phosphate phosphatase sequences. The alignment was generated with SBAL[2] using secondary structure prediction obtained with PSIPRED[3]. Helical structure is indicated in green, beta-strands are shown in red and cysteine residues are highlighted yellow. The domain topology is indicated in the top lines. Active site aspartate residues are highlighted by '*'.

Supplementary Figure S2

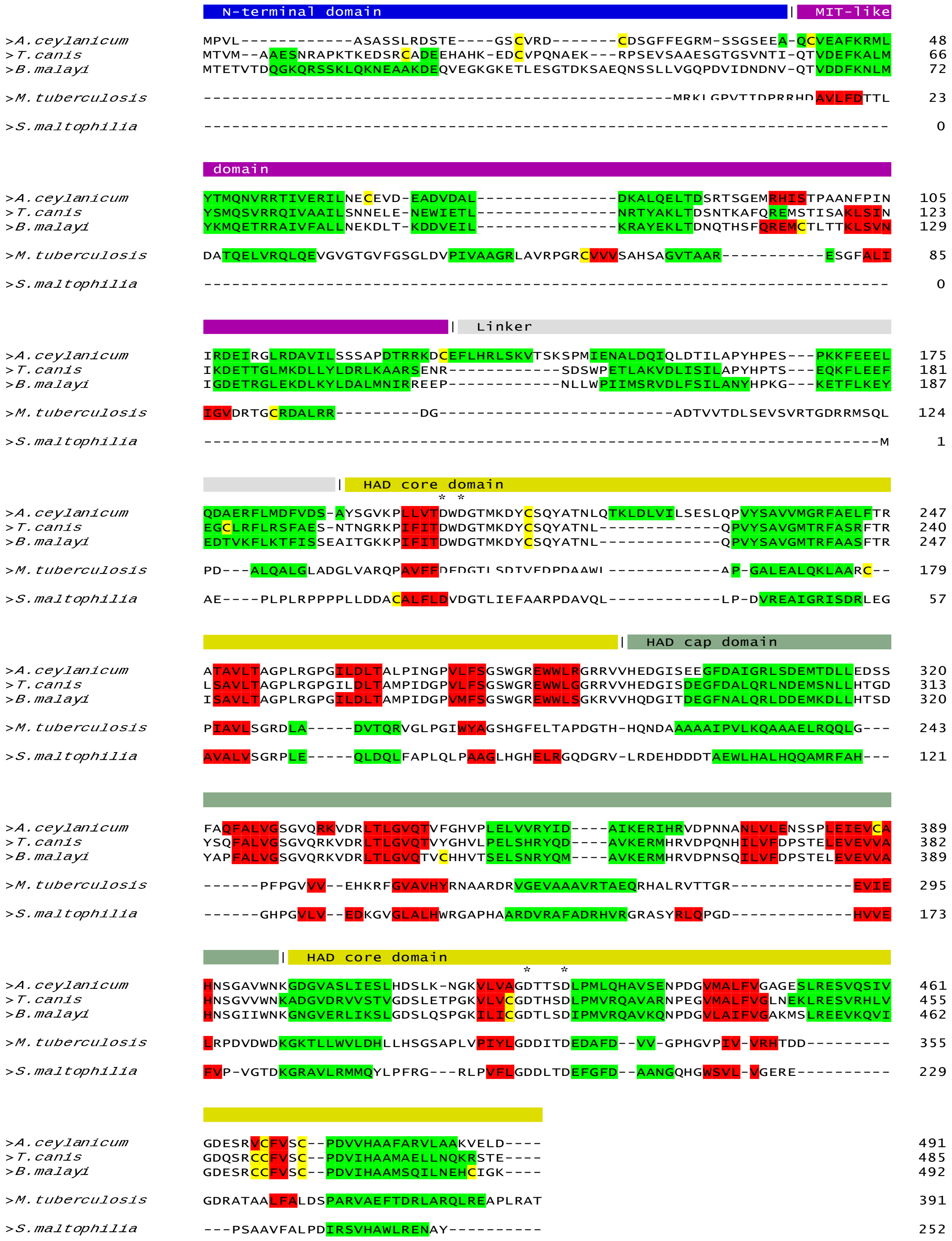

S3 Validation of recombinant proteins by SDS-PAGE

Supplementary Figure S2 (below) shows a 12% denaturing reducing SDS-PAGE of the final purified recombinant proteins used in this study with exception of TPP from *B. malayi*; for *Bmal*-TPP, please see the Supplementary Information of[1].

Supplementary Figure S3

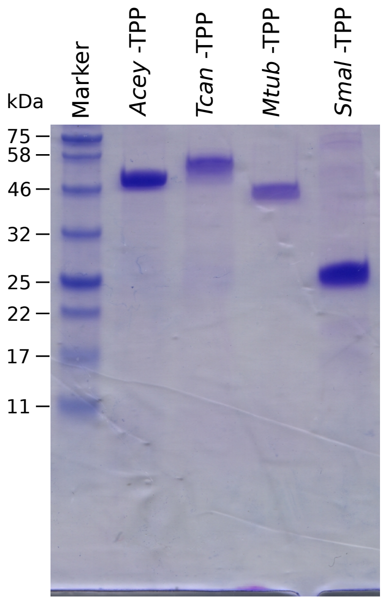

S4 Validation of secondary structure content by circular dichroic spectroscopy

Supplementary Figure S3 (below) shows the CD spectra of the final purified recombinant proteins used in this study. Spectra were recorded in buffer containing 10 mM NaH_2PO_4, pH 8.0 at protein concentrations between 0.08 and 0.1 mg ml^{-1} using a Jasco J715 Spectropolarimeter and converted to mean residue ellipticity with the software ACDP[4].

Supplementary Figure S4

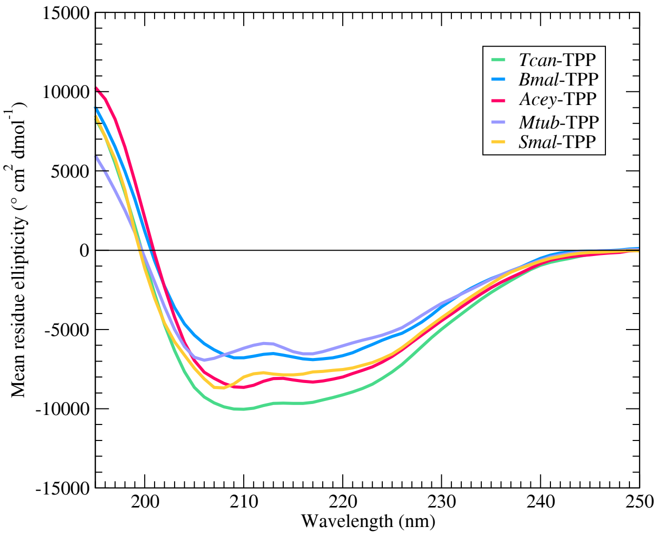

S5 Validation of recombinant proteins by MS fingerprinting

50 µg of each sample in 50 µL was reduced with 10 mM DTT heated at 60°C for 1 hr. Then, samples were alkylated with 22 mM iodoacetamide for 30 min in the dark. For limited proteolysis, 2 µg of trypsin were added to each sample (1:25 ratio trypsin:protein). After digestion, 4 µg aliquot was taken, dried and reconstituted into 20 µl loading buffer prior to analysis.

Prior to nanoflow electrospray MS data acquisition, 10 µl of sample were loaded onto a peptide trap column for pre-concentration and desalting with 0.1% formic acid, 2% acetonitrile, at 10 µl min^{-1} for 5 minutes. Peptides were eluted from the column using a linear solvent gradient (95:5 → 5:95 H_2O:acetonitrile ratio, + 0.1% formic acid) with constant flow (500 nl min^{-1}) over an 80 min period. The LC eluent was subjected to positive ion nanoflow electrospray MS analysis by acquiring a TOF-MS survey scan with the ten largest multiply charged ions (counts >150) sequentially subjected to MS/MS analysis. MS/MS spectra were accumulated for 200 ms with rolling collision energy.

Supplementary Table S4 (below) summarises the sequence coverage observed in MS fingerprinting of the five samples.

Supplementary Table S5

Protein	Sequence coverage in MS fingerprinting
Acey-TPP	26%
Bmal-TPP	57%
Tcan-TPP	60%
Mtub-TPP	70%
Smal-TPP	79%

References

1. Cross, M. *et al.* Probing function and structure of trehalose-6-phosphate phosphatases from pathogenic organisms suggests distinct molecular groupings. *FASEB J.* **31,** 920–926 (2017).
2. Wang, C. K. *et al.* SBAL: a practical tool to generate and edit structure-based amino acid sequence alignments. *Bioinformatics* **28,** 1026–1027 (2012).
3. Bryson, K. *et al.* Protein structure prediction servers at University College London. *Nucleic Acids Res.* **33,** W36–W38 (2005).
4. Hofmann, A. *ACDP* – a Java application for data processing and analysis of protein circular dichroism spectra. *J. Appl. Crystallogr.* **42,** 137–139 (2009).
